# Histological and Physiological Study of the Effects of Biostimulants and Plant Growth Stimulants in *Viburnum opulus* ‘Roseum’

**DOI:** 10.3390/plants13111446

**Published:** 2024-05-23

**Authors:** Dezső Kovács, Katalin Horotán, László Orlóci, Marianna Makádi, István Mosonyi, Magdolna Sütöri-Diószegi, Szilvia Kisvarga

**Affiliations:** 1 Institute of Landscape Architecture, Urban Planning and Garden Art, Hungarian University of Agriculture and Life Sciences (MATE), 1223 Budapest, Hungary; kovacsdezso.zsztgy@gmail.com (D.K.); orloci.laszlo@uni-mate.hu (L.O.); mosonyi.istvan.daniel@uni-mate.hu (I.M.); sutorine.dioszegi.magdolna@uni-mate.hu (M.S.-D.); kisvarga.szilvia@uni-mate.hu (S.K.); 2 Institute of Biology, Eszterházy Károly Catholic University, 3300 Eger, Hungary; 3 Research Institute of Nyíregyháza, IAREF, University of Debrecen, 4400 Nyíregyháza, Hungary; makadim@agr.unideb.hu

**Keywords:** biostimulants, ornamental, woody plants, shrubs, green surface, sustainable, Viburnum, physiology, growth, rhizosphere

## Abstract

Biostimulants and other plant growth promoters can provide an effective solution to the challenge of urbanisation and climate change. *Viburnum opulus* ‘Roseum’ is a globally popular deciduous shrub species that can be made more resistant to urban influences by using natural growth-promoting substances. In our study, we investigated the effects of growth promoters Kelpak^®^, Bistep and Yeald Plus on the species, both histologically and physiologically (proline stress hormone measurement). Our measurements were complemented using the analysis of rhizosphere alkaline phosphatase, β-glucosidase and β-glucosaminidase enzymes, to obtain a more complete picture of the combined effect of biostimulants and species. We found that the Bistep biostimulant had an outstanding effect on the leaf tissue culture results of the variety. The transpiration and evapotranspiration findings also confirmed the efficacy of biostimulants. In the case of POD activity and rhizosphere enzyme measurements, Bistep and Yeald Plus obtained statistically higher values than the control group. Kelpak produced better results than the control group in several measurements (alkaline phosphatase levels; evapotranspiration results), but in other cases it resulted in lower values than the control treatment. The use of Bistep and Yeald Plus can greatly assist growers in the cultivation of *V. opulus* ‘Roseum’ in an urban environment.

## 1. Introduction

Continuing to secure and maximise crop yields under the threat of climate change is a major challenge for the agricultural sector, which is under further pressure from demographic projections (9.5 billion global population by 2050) [1]. According to current projections, global agricultural production will decrease by 50% and 30% respectively due to abiotic and biotic stresses [2]. To reduce and counteract this, several technological innovations have been proposed over the past three decades to enhance the sustainability of agricultural production systems, with the aim of reducing the use of synthetic agricultural chemicals (pesticides, fertilisers). Biostimulants have emerged as an alternative to replace these, and are often used in several horticultural sectors, including ornamental horticulture. In the latter sector, the application is aimed at increasing the vitality and resistance of plants while achieving commercial standards, which is a prerequisite for producing quality commodities [3,4]

Based on what has been described so far, the use of natural plant biostimulants (PBs) is a promising and environmentally friendly trend that is gaining increasing importance [5]. PBs also promote and enhance flowering, growth, crop setting, yield and nutrient use efficiency (NUE), in addition to their ability to improve tolerance to environmental factors [6,7,8,9]. The benefits provided also have implications for the use of biostimulants, with their use projected to increase by 10.4% per year globally until 2030 [10].

Plant biostimulants from natural products are attracting increasing interest among researchers, farmers and industrial companies as an effective tool to increase crop yields [11]. They can be derived from various organic materials and may contain humic substances, complex organic substances, useful chemical elements, peptides and amino acids, inorganic salts, seaweed extracts, chitin and chitosan derivatives, transpirants, and other nitrogen-containing substances. The use of biostimulants in plants leads to higher nutrient content in their tissues and positive metabolic changes [12]. Biostimulants are substances that incorporate one or more substances and/or microorganisms that promote plant nutrient uptake and use efficiency, increasing the tolerance of plants to abiotic/biotic stresses, and, when applied in small quantities, improve crop quality or morphological and physiological characteristics [13]. In addition to these benefits, biostimulants can enhance the activity of rhizosphere microbes and soil enzymes, the production of hormones and/or growth regulators in soil and plants, and photosynthetic processes [14]. The mechanisms of the action of biostimulants are often unknown and difficult to identify, as they are usually derived from more complex sources containing multiple bioactive compounds that may collectively combine to contribute to specific effects on plants.

Biostimulants can also positively influence other plant traits beyond the stress response. The application of *Ascophyllum nodosum* algae as biostimulants has been shown to increase photosynthetic rate, stomatal conductance, and transpiration in treated plants, as well as to increase stress tolerance [15]. In addition, the beneficial effects of biostimulants on leaf tissue and the intestinal tissue system are also observed [16]; several types of biostimulants, including humic acids, have been shown to possess cell wall strengthening effects [17]. Pylak et al. [18] have shown that the low molecular weight of fulvic acids allows them to penetrate tissues through membrane pores and form complexes with cations that help transport nutrients into the cell.

Sharma et al. [19] have shown that marine algal extracts stimulate chlorophyll synthesis due to their high hormone levels. Higher chlorophyll levels have been observed when algal extracts were applied to potato plants [20]. Apart from algae extracts, other types of biostimulants can also induce an increase in chlorophyll storage, which has been shown to induce significant chlorophyll growth [21]. Nardi and his colleagues [22] stated that humic acids can affect photosynthesis, resulting in an increase in shoot and root mass in the cultivar studied. On the other hand, a direct relationship between chlorophyll and carotenoid content has been demonstrated [23].

Plant responses to abiotic stresses are the result of the interaction between different signalling molecules and hormonal balance, which exert their effects by altering metabolic and gene expression levels. These effects could explain that the studied biostimulants significantly reduced the levels of stress enzymes, including peroxidase (POD) and superoxide dismutase [24,25]. Biostimulants can increase biomass, photosynthetic pigments and modified chlorophyll fluorescence-related parameters [26]. A correlation between stress enzymes and proline levels has also been observed [27], which may also be of interest for specific measurements, as increased proline levels are associated with increased transpiration and evapotranspiration results [28]. The transpiration rate showed a decreasing trend during the plant growth period, with increasing leaf temperature and stomatal diffusion resistance [29]. Proline accumulation has been shown in plants exposed to various abiotic stresses and, according to Tripathi and Gaur [30], proline protects cells by scavenging free radicals during oxidative stress caused by heavy metals. The literature suggests that proline protects plants by acting as a cellular osmotic regulator between the cytoplasm and the vacuole and detoxifies reactive oxygen species (ROS), thus ensuring membrane integrity and stabilising antioxidant enzymes [31].

In the case of ornamental plants, several studies have investigated how biostimulants affect them under different conditions, for example in urban plantations. Akensous and colleagues [32] have studied plant growth-promoting rhizobacteria (PGPR) and biostimulants extracted from compost materials that can increase drought tolerance or morphological traits [33]. The latter effects have been described in several herbaceous ornamental plant species grown in containers [34]. Zhang in his study [35] found that *Festuca arundinacea*, also used as an ornamental plant, enhances the defensive response to heat stress through lipid conversion. Annual ornamental plants were treated with a humic acid-based biostimulant, which improved the plants’ tolerance to thermal stress and their nutrient uptake from the soil with a concomitant reduction in fertiliser application requirements, consequently reducing environmental stress and improving overall plant quality. The acidic pH peat used as a medium for the plants was not suitable for β-glucosidase and β-glucosaminase enzymes, which are very sensitive to soil pH [36]. Another possible reason could be the involvement of the enzyme β-glucosaminase in maintaining plant health, which plays an important role in the protection against pathogenic fungi and animal pests, as described by Parham and Deng [37].

Their application increases leaf and flower number as well as chlorophyll content and net photosynthesis, parameters that can be of particular importance in ornamental plant production and urban applications [38]. Seaweed extracts and humic and fulvic acids, used as biostimulants, can also be a solution to increase the flower number in woody-stemmed species with ornamental flowers and to increase vase life in cut flowers [39,40,41]. The use of a combination of different types of biostimulants and fertilisers may also be a good option in ornamental plant applications [42,43]. In ornamental shrub applications, biostimulant improved root morphology, increased leaf number and thus leaf area and dry biomass accumulation [44].

*Viburnum opulus* L. belongs to the plant family *Caprifoliaceae* [45] and is a valuable ornamental, medicinal and food plant. The basic species *V. opulus* shrub is common in natural habitats in Western and Central Europe, Asia, Caucasus and Asia Minor [46,47,48]. It is a fast-growing deciduous shrub up to 4–5 m tall. The leaves are transverse, three-lobed, rounded at the base and have roughly serrated margins. The upper part of the leaves is bare and dark green, and the leaf filaments are lighter and slightly hairy with star-shaped hairs. Leaves develop with the flowers, then turn scarlet-reddish-purple [49]. White flowers develop in cups 4–11 cm in diameter at the top of the stem. Each flower is composed of an outer ring of large sterile flowers and an inner ring of small fertile flowers. The ornamental cultivar ‘Roseum’ has only sterile flower spikes, which give it a snowball-like appearance [50].

In their study, Kajszczak et al. [48] and Levent Altun et al. [51] explain that the main publications on *V. opulus* so far are on its morphology, botany and medicinal uses, with the main focus on the fruits of the species [51] and its cultivation processes as a fruit [52]. However, the species is an important and frequently used ornamental shrub, especially because of its varieties, and has been little studied from this point of view. ‘Roseum’ (syn. ‘Sterilis’, ‘Snowball’), also a little studied ornamental shrub, although a long established and popular species, is a traditional ornamental plant in Hungary and Europe. This choice was supported by the fact that the species was known as ‘Sambucus Rose’ as early as the 16th century, so its conservation and maintenance as a heritage species may be important for the future. It is still a commonly used cultivar, which prefers a temperate climate, making it often difficult to maintain and keep healthy in urban areas. The use of growth regulators and biostimulants during cultivation and rearing can be an important and environmentally friendly method of successful plant production.

Research into the use of growth regulators that can be used in the horticultural production of future varieties may be important, including the testing and incorporation of biostimulants into horticultural cultivation. Therefore, the aim of treating *V. opulus* ‘Roseum’ as a popular urban shrub with plant growth regulators is to demonstrate their effects on the variety from several aspects, including morphological, histological and physiological aspects, and to find a biostimulant for the variety with the potential for effective and favourable utilisation.

Due to the lack of literature in this field, the aim of our study was to contribute (i) to the knowledge about this species from an ornamental horticultural point of view, and (ii) the rising importance and expansion of sustainable horticulture and agriculture, which will also contribute to the knowledge of the potential urban uses of this species.

## 2. Results

### 2.1. Histological Results

**Figure 1:**
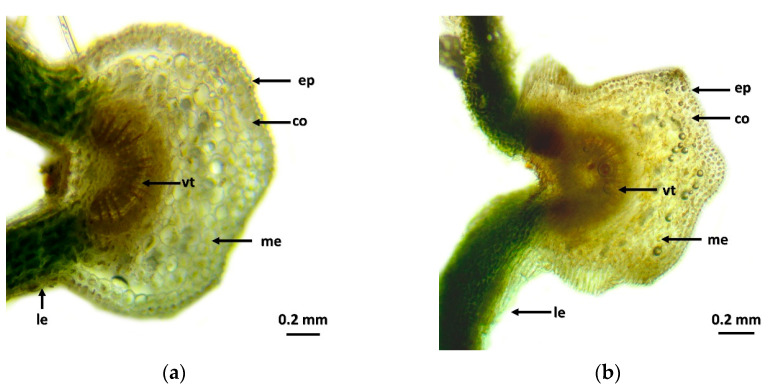

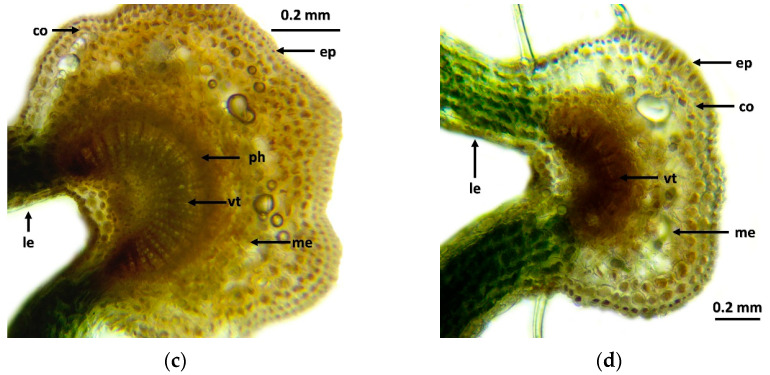
Microscopic images of leaf tissue and transport rays in *V. opulus* ‘Roseum’ as a result of treatments. (**a**) Control; (**b**) Kelpak; (**c**) Yeald Plus; (**d**) Bistep. The abbreviations shown in the pictures mean the following: ep—epidermis; co—collenchyma; vt—vascular tissue; le—leaflet; me—mesophyll, ph—phloem.

The microscopic images show that the effects of the treatments on the transport tissues are clearly visible (Figure 1). In the control group (Figure 1a), the epidermis cells are homogeneous, the cell walls are intact, the transport cells are formed and the intestinal tissue cells are healthy. In the case of the Kelpak^®^ biostimulant (Figure 1b), there was no detectable effect on leaf tissue, thus correlating with other results of Kelpak^®^ biostimulant-treated plants. The leaf cross-section shows the same shape as the control group. In Yeald Plus (Figure 1c) and Bistep (Figure 1d), more significant differences in the transport tissues were observed compared to the control; in addition, the epidermis cells were thickened, the cell lines were uniform and strong, and the collenchyma cells were homogeneous and delimited. The transport tissue is regularly outlined by beams and secondary thickening, with individual areas being more distinct (woody and collenchyma) than in the Kelpak^®^ and control groups.

### 2.2. Physiological Results

#### 2.2.1. Transpiration and Evapotranspiration

**Figure 2:**
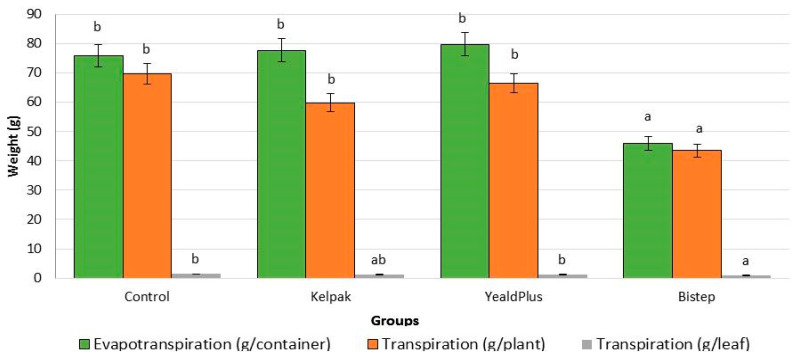
Evapotranspiration and transpiration in *V*. *opulus* ‘Roseum’ cultivars under the influence of bistimulants and growth regulators. To compare differences, ANOVA and one-way ANOVA with Duncan test were performed (*p* < 0.05). Different letters indicate different statistical groups.

Measuring and accurately determining the transpiration and evapotranspiration values will provide a more nuanced picture when investigating the effects of growth regulators (Figure 2). Based on the measured results, the evapotranspiration of the control group, Kelpak^®^ (3%) and Yeald Plus (4.8%) were statically indistinguishable, followed by the transpiration data. However, for the Bistep biostimulant, both the evapotranspiration and transpiration data show a significant decrease. These values indicate a decrease in evapotranspiration for the Bistep biostimulant, which can be described as an indication of improved water balance in the soil and the plant. A strong correlation can be observed when these results are combined with the transpiration results per leaf.

#### 2.2.2. Chlorophyll and Carotenoid Content

**Figure 3:**
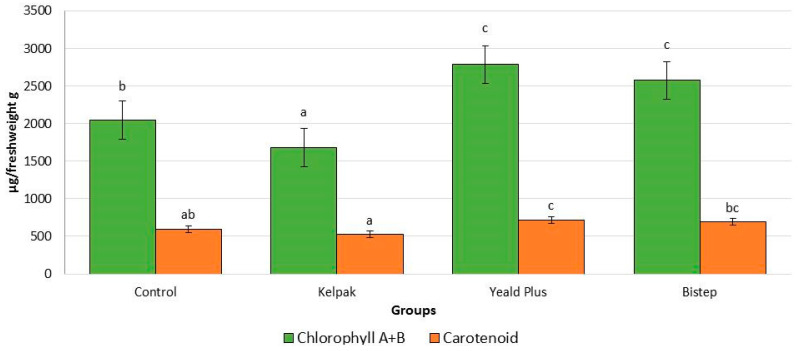
Chlorophyll (A + B) and carotenoid content in *V. opulus* ‘Roseum’ cultivars under the influence of growth regulators and bistimulants. To compare differences, ANOVA and one-way ANOVA with Duncan test were performed (*p* < 0.05). Different letters indicate different statistical groups.

Significant differences were observed for both the chlorophyll and carotenoid content of the biostimulants tested (Figure 3). The chlorophyll content of the control group was significantly higher than that of the Kelpak^®^-treated group (18%) which did not exceed the other two biostimulants, and Kelpak^®^ produced the lowest chlorophyll results. The growth regulators Yeald Plus (36%) and Bistep (20.5%) had statistically higher chlorophyll contents than the control group.

Such a strong difference was not observed for carotenoid content; however, the values measured in the control group show a statistically significant difference only with the growth regulator Yeald Plus (11.2% higher). The lowest carotenoid content was found for the Kelpak^®^ biostimulant (13.5% lower), which is significantly lower than the values measured in the other groups.

#### 2.2.3. Proline Level

**Figure 4:**
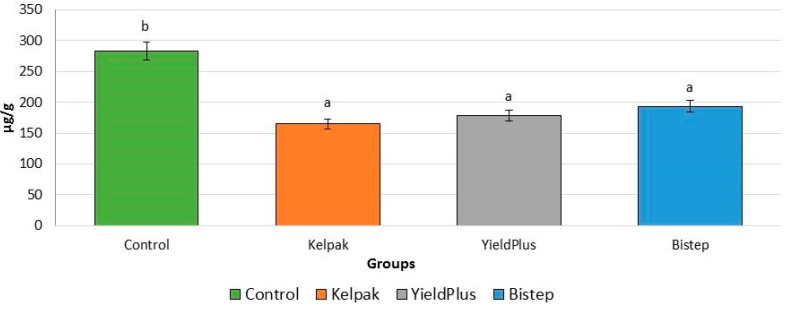
Proline level in *V. opulus* ‘Roseum’ cultivars under the influence of bistimulants and growth regulators. To compare differences, ANOVA and one-way ANOVA with Duncan test were performed (*p* < 0.05). Different letters indicate different statistical groups.

Figure 4 summarises the results of the proline level measurements, showing significant differences between the control group and the treated groups. All treated groups showed significantly lower levels compared to the control group. There were no statistical differences between treatments. The lowest proline level was measured with the Kelpak^®^ biostimulant (58.2% lower proline level).

#### 2.2.4. Peroxidase Enzyme

**Figure 5:**
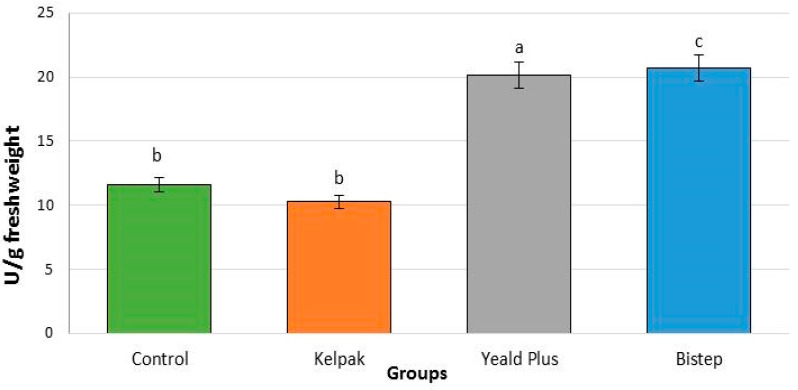
Peroxidase enzyme activity level in *V. opulus* ’Roseum’ cultivars under the influence of growth regulators and biostimulants. To compare differences, one-way ANOVA with Duncan test was performed (*p* < 0.05). Different letters indicate different statistical groups.

Plants with higher antioxidant levels are more resistant to oxidative stress, and these enzymes are considered biomarkers of biotic and abiotic stress effects [53]. In the *V. opulus* ‘Roseum’ cultivar, the POD enzymes present in the control group did not show a significant difference compared to the Kelpak^®^ biostimulant (12.9%) (Figure 5). In the Yeald Plus biostimulant, the POD value was significantly increased (73.5%), while the highest value was detected in the Bistep-treated group (78.6%).

#### 2.2.5. Rhizosphere Enzyme Activities

The soil life associated with microorganisms in the rhizosphere, the environment of the root system of *V. opulus* ‘Roseum’, can be an important parameter for the study of the effects of biostimulants and mineral elements on plant and soil life.

**Figure 6:**
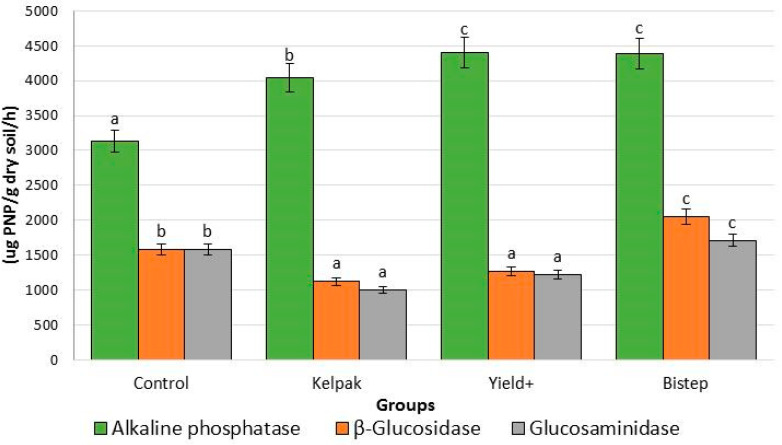
Activity of some enzymes of the rhizosphere during the treatment of *V. opulus* ‘Roseum’ with bistimulants. To compare differences, ANOVA and one-way ANOVA with Duncan test were performed (*p* < 0.05). Different letters indicate different statistical groups.

The alkaline phosphatase enzyme level was lowest in the control group, while it was significantly higher in all treated groups (Figure 6). However, the β-glucosidase enzyme levels were significantly lower in the Kelpak^®^- (29.2%) and Yeald Plus-treated groups (20.1%) compared to the control group. The highest enzyme level was measured for Bistep (77.3% higher), which represents a statistically different group compared to the other treatments. The levels and significance data for the enzyme β-glucosidase and β-glucosaminidase showed a similar pattern in the treatment with Kelpak^®^. Kelpak^®^ resulted in the lowest levels of these enzymes. The highest β-glucosidase and β-glucosaminidase activities were measured in the Bistep treatment.

## 3. Discussion

There are several ornamental plant species that have been very popular and widely used since their first commercialisation, but their tolerance needs to be supported at several levels as environmental conditions change. The traditional species *V. opulus* ‘Roseum’ is a conspicuous ornamental shrub, both in its habit and inflorescence, as described by Kajszczak et al. [50]. The species can currently be grown safely in urban conditions, but in a few years its cultivation may become endangered and as it is less resistant to the changing urban environment. To prevent this, treatment with biostimulants and other growth regulators and plant conditioners that do not cause environmental stress could be a solution.

We have been running experiments with three biostimulant products named Bistep, Yeald Plus and Kelpak^®^. Bistep, also known as Ferbanat L, is a biostimulant containing humic and fulvic acids [54,55]. Yeald Plus is a rooting stimulant containing a growth regulator in the form of zinc ammonium acetate [56]. Kelpak^®^ is a biostimulant containing marine algae extracts with the crushed parts of *Ecklonia maxima* [57].

The plant growth regulators had a beneficial effect on leaf tissue condition, both epidermal and intestinal, as discussed by Soppelsa and et al. [16]. The effect of Bistep and Yeald Plus was particularly effective, whereas there was no outstanding difference for Kelpak^®^ compared to the control group [58]. These results also relate to weight gain outcomes, which follow a similar trend. It can be said that the application of Bistep and Yeald Plus strengthened the transport tissues. In parallel, a decrease in transpiration and evapotranspiration values can be explained. This is in agreement with the measurements of Conselvan et al. [17] that humic acid-based biostimulants have cell wall strengthening effects. Consistent with the findings of Pylak et al. [18], the results on histological measurements can be explained for the humic acid-containing Bistep biostimulant, as Kelpak^®^ resulted in the lowest chlorophyll and carotenoid content. Kelpak was also found to have a negative effect in terms of histological results, which cannot be correlated with the results of Kularathne et al. [59], as the plants were in a strong cell division and elongation phase during the growing period under treatment.

The findings of Wadas et al. [20] on *V. opulus* ‘Roseum’ also provide evidence that humic acids can affect both respiration and photosynthesis, as the application of humic acid-containing Bistep significantly increased both chlorophyll and carotenoid content. Nardi et al. [22] found a similar result and also found higher rates of photosynthesis increase compared to the control group. On the other hand, a direct relationship between chlorophyll and carotenoid content was found, as their levels increase or decrease in the same proportion under the influence of growth regulators, as shown by the results of Ngoroyemoto et al. [23].

Assuming that the use of biostimulants and other plant growth regulators reduces the stress state of the plant, we completed our series of measurements by determining the proline levels and peroxidase enzyme activity levels associated with stress response. The proline level produced by the control group was significantly higher than in the treated groups, where *V. opulus* ‘Roseum’ was found to be beneficially affected by biostimulants, with the biostimulant Bistep being the most prominent. However, peroxidase enzyme activity indicated that stress levels were significantly increased using Yeald Plus and Bistep and reached statistically higher values than in the control group. As peroxidase enzyme activity increased, lower proline levels were detected, confirming the claim of Ozden et al. [27] that this was observed in *V. opulus* ‘Roseum’ in both control and treated plants. The results of Rajametov et al. [28] are in agreement with our results on measured respiration parameters; evapotranspiration, transpiration and evaporation per leaf were the highest in the control group plants, while the lowest values were found in the Bistep biostimulant-treated groups. The decrease in transpiration rate, in comparison with the results of Surendar et al. [29], suggests that at lower transpiration values, the stress cell resistance is higher. Proline regulates cellular changes by preventing water loss through osmotic regulation, which, in addition to higher proline levels, may explain the higher transpiration values in the culture strain studied. It was hypothesised that the increase in proline levels indicates higher stress levels following the results of Bohnert and Jensen [31], which may be explained by the beneficial effects of biostimulants on stress responses.

Rhizosphere is a special environment where strong communication takes place between plant and microbes [60]. Since biostimulants and plant growth regulators can affect the whole plant, including its root system and rhizosphere, it was considered essential to conduct soil microbiological studies. We proposed increasing enzyme activities in the rhizosphere of the studied plant variety because alkaline phosphatase, β-glucosidase and β-glucosaminidase enzymes help plants in the uptake of macroelements, but significant differences were found among the effects of biostimulants and enzyme activities. In the case of alkaline phosphatase activity, which is produced only by microbes, significant increases were found in the treatments compared to the control one. All the applied biostimulants contained microelements and/or organic matter, and nitrogen but not phosphorous (P); therefore, the increased P demand of plants resulted in an increased alkaline phosphatase activity in the rhizosphere [61]. The addition of nutrients (e.g., nitrogen) to the soil with the biostimulants could decrease the β-glucosidase and β-glucosaminidase activities [62], as it was found in the case of Kelpak and Yield Plus treatments. However, the main effects of humic substances, which have also been described for Bistep, are increased root mass and improved morphological properties, increased nutrient uptake and utilisation efficiency, and consequently improved yields [54]. These effects of Bistep could be strengthened by the increased enzyme activities in the rhizosphere.

Based on the results measured and evaluated during the studies, it can be concluded that the *V. opulus* ‘Roseum’ cultivar can be promoted and its wide use can be protected in the future through the application of biostimulants. Of the three biostimulants used, Bistep has shown outstanding results and its use is recommended for the cultivation of the variety.

## 4. Materials and Methods

### 4.1. Biostimulants Used for Treatment

#### 4.1.1. Bistep

Bistep, internationally known as Ferbanat L plant conditioner, is a naturally derived liquid nano-fertiliser specialised in stress management. It contains micro-humusates coupled with microelements and hydrologically active natural substances. It contains a combination of humic acid and fulvic acid, which stimulate root development. Its use increases the amount of soil humus, since it has a 2-year after-effect after use thanks to the micro-organisms. The manufacturer guarantees that the use of the product reduces the use of fertilisers by about 50% and also the need for pesticides. It is most effective when applied at appropriate intervals from the very first phenophase of the plant.

To better understand the mechanism of action of Bistep, a detailed knowledge of its constituents is necessary. Humic acids, described as dark-coloured, heterogeneous aggregates of organic matter, are the result of microbiotic metabolism and can be characterised as one of the most abundant organic substances, highly resistant to various environmental stresses [63]. The traditional, albeit nowadays criticised, classification of this material distinguishes three groups: humic acids, the water-insoluble part; humic acids (or HAs), which are soluble in pH > 2; and fulvic acids (also FAs), which are soluble in water [64]. This classical definition is still present in the older scientific literature, but chemically HS is nothing more than the product of saponification reactions from soils and sediments through alkaline extraction [14]. However outdated, this division is the most commonly used in all the literature reviewed, as it allows a meaningful distinction between the products, i.e., a clear distinction between humic acid-based and fulvic acid-based products, and is therefore appropriate [65]. Fulvic acids (FAs) improve the structure and fertility of heterogeneous textured soils and play a crucial role in increasing biomass and yields in crop production [66]. Humic acid and fulvic acids together convert minerals into organic compounds that are readily taken up by plants [64]. The main effects of humic substances, which have also been described for Bistep, are increased root mass and improved morphological properties, increased nutrient uptake and utilisation efficiency, and consequently improved yields [54].

#### 4.1.2. Kelpak^®^

Kelpak^®^ is made from *Ecklonia maxima* dark brown algae, which grow in the cold waters off the west coast of South Africa. This fast-growing algae species is characterised by elevated levels of auxin and cytokinin-like substances. Kelpak^®^ contains plant cells extracted from freshly cut and crushed plants without heating, freezing or the use of chemicals [57]. Marine algae extracts are generally the sum of the products of aqueous or solvent extraction and hydrolysis of the biomass of algal species from the genera *Ascophyllum, Ecklonia, Macrocystis* and *Durvillea* [67,68]. Among these, preparations containing extracts of algal species *Ecklonia maxima* and *Ascophyllum nodosum* are the most widely used [69], e.g., Kelpak^®^ and Goëmar BM 86^®^ biostimulants [70].

The production methods are not standardised but are patented, and eight extraction methods are currently in use [71]. Seaweed extracts are widely used in agricultural and organic farming, and their use in the ornamental horticulture sector is also significant, as they have a positive effect on plant quality by promoting plant development. Their widespread use started in the mid-2000s [72]. The degree of effectiveness of algal extracts depends on two factors: one is the hormone level of the plants and the other is the number of micronutrients (mainly cytokines) present in the crude extract [59].

#### 4.1.3. Yeald Plus

Yield Plus is marketed specifically as a rooting promoter [56] and is produced by De Sangose Ltd. in the UK. Its application can achieve up to 30–35% root mass increase in two weeks [73]. Its application is facilitated by the plant’s ability to take it up and utilise it adequately at root and leaf temperatures even at lower temperatures (+5–7 °C), thus providing flexibility of use, further supported by its good root and leaf surface utilisation. For transplanted vegetable crops, dipping the roots in a 0.05–0.1% solution before transplanting and foliar fertilisation at 1–1.5 litres/ha after transplanting may be recommended. As a generally accepted rule of application, the younger the plant, the lower the dose [74].

Yield Plus owes its effectiveness to the ammonium acetate form of zinc, a proprietary technology. Composition: 6% nitrogen, 5% zinc, 1% potassium peroxide, 0.03% boron, 0.25% copper, 0.25% iron, 0.25% manganese and 0.001% molybdenum. In terms of its effects, it stimulates auxin synthesis, supports root development, improves water and nutrient uptake, increases chlorophyll levels and accelerates carbohydrate metabolism [73]. Based on the literature, its use in nursery production of woody stem plants is not yet widespread (Table 1).

### 4.2. Plant Material Used in the Experiment

**Figure 7:**
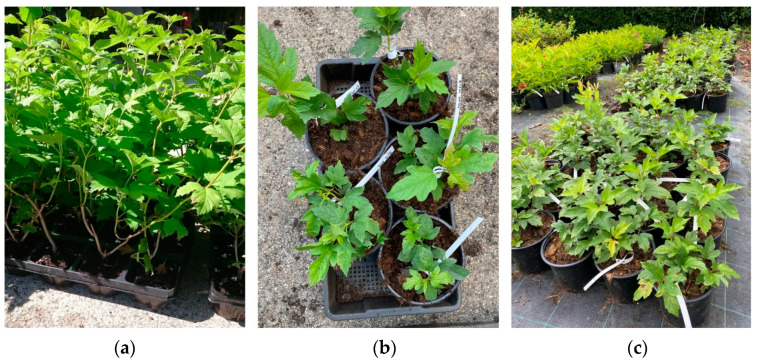
*V. opulus* ‘Roseum’ experimental specimens at (**a**) basal stand before treatment and potting (**b**) potted and first treated plants before transport to the nursery (**c**) condition after last treatment at the nursery (Kovács, 2023).

Plant selection was based on a complex set of criteria, which included choosing plants that were widely available in woody nurseries. In order to schedule the trials and monitor development, the plant had to be selected from a range of shrubby plants that have long been used and favoured in private gardens in municipalities and in urban green spaces. The cultivar *V. opulus* ‘Roseum’ has these characteristics. The shrubs were propagated in our nursery in Hungary (Figure 7).

### 4.3. Planting Parameters

The plants in the experiment were planted in 1.5 litre containers. The composition of the planting medium consisted of 30% milled black peat (pH: 6.24; humus content: 17.89%) and 70% milled white peat (pH: 5.81; humus content: 25.58%) with a particle size of 10–30 mm and EC levels ranging from 2.0 to 4.0.

The measurements were made with 25 individuals in 5 replicates. Plants were uniformly top watered with sprinkler heads. No pruning was performed during their development. Plant protection treatments were applied only and exclusively against aphid damage, using Mospilan 20SG (manufacturer and distributor Sumi agro Hungary Kft.).

The first treatment was applied at the same time as planting; the planting was carried out during the whole week of 1 May each year, and thereafter repeated spraying was carried out once every 5 weeks until the shoots stopped growing. The end of July is the end of shoot growth in the Hungarian climate. Accordingly, the plants received a total of 3 treatments per season. The concentrate rates applied were as follows:

- Kelpak^®^ 0.4% solution;
- Yeald Plus 0.3% solution;
- Bistep 0,5% solution;
- Control group received only water at the same time as the treatments.

### 4.4. Histological Measurements

Based on the literature background, the agents used and their active ingredients can modify a number of morphological parameters, so it was considered important to assess the treated plants at tissue level. For this purpose, leaf cross-sectional images were taken and the leaf material was collected in the 5th week after the last treatment. Leaf tissue samples were cleaned with distilled water and mechanically sectioned with a blade, and fresh sections were used to eliminate any distortion that might occur during fixation and to obtain as accurate a picture of changes as possible.

A Euromex bScope BS.1153-PLi biological microscope with a compatible camera (Levenhuk m1400 plus) was used for the study. Due to the nature of the sections, PLi 4/0.1 lenses were used, which provided a magnification of 40×. The eyepiece was of type and size WF120×/20. Samples were unstained. The images were post-corrected using GIMP 2.10 (property of Spencer Kimball, Peter Mattis).

### 4.5. Physiological Measurements

Measurement of leaf transpiration and calculation of leaf conductance to water vapour are important in almost all studies of plant water relations [75]. To assess the physio-logical status, essential for a complex study of the effects of growth stimulants, transpiration and evapotranspiration parameters were recorded. To carry out the survey, the tanks were filled evenly with water the day before. In order to measure only evaporation through the canopy, the tanks were covered with waterproof material to prevent other water loss from the canopy. Measurements were taken the following morning and evening using this lysimeter. The fresh weight change, the combination of plant growth and water status changes can be calculated from evapotranspiration and water uptake. The system consists of two contacting containers filled with nutrient solution: each is placed on an electronic balance. One of these vessels carries the plant and is connected to the other by a flexible tube. Water uptake in each vessel reduces the solution level equally. The mass loss of the vessel without the plant gives the rate of water uptake, the total mass loss on both scales gives transpiration. The difference between the two data gave the evaporation rate, i.e., the amount of water evaporated through the canopy and the surface of the medium, as described by Van Leperen et al. [76], which also showed the rate of transpiration and evapotranspiration.

### 4.6. Chlorophyll and Carotenoid Content Measurement

Chlorophyll and carotenoid content were determined according to the method of Arnon [77]. Accordingly, a 150 mg fresh weight plant sample, weighed to 3 decimal places (grams), was homogenised on an analytical balance (Explorer Pro 64, OHAUS Europe, Nänikon, Switzerland) in a rubbing flask cooled to below 4 °C with quartz particles, in the presence of a knife-edge of Na_2_CO_3_ in 80 *v/v*% acetone. The homogenised samples were filled to 10 mL final volume with 80 *v/v*% acetone and centrifuged at 4 °C at 1000 rpm (5418 R, Eppendorf AG., Hamburg, Germany) for 10 min.

The absorbance of the supernatant was determined using a spectrophotometer (GeneSys VIS-10, Thermo Fisher Scientific Inc., Waltham, MA, USA) at wavelengths λ1 = 663, λ2 = 644, λ3 = 480 nm, and the chlorophyll content was calculated from the values obtained using the following formula:

µg chlorophyll/g feed = (20.2 × A644 + 8.02 × A663) × V/w

where:

- Ax—absorbance at a given wavelength;
- V—final volume of the sample;
- w—the sampled and homogenised plant fresh weight.

For the determination of dry matter content, the plant samples were dried at 80 °C for 24 h before drying, and afterwards their mass was measured on an analytical balance (Explorer Pro 64, OHAUS Europe, Switzerland) to 3 decimal places.

### 4.7. Peroxidase Enzyme Activity Measurement

Peroxidase (POD) activity was measured spectrophotometrically (λ = 460 nm) in the presence of H_2_O_2_ substrate in the method of Shannon et al. [78] using orthodianisidine chromogenic reagent (molar absorption coefficient for oxidised orthodianisidine: ε = 11.3 mM^−1^ cm^−1^). For the measurement, samples were prepared from 300 mg of fresh plant tissue in a rubbing flask cooled to below 4 °C using quartz sand and phosphate buffer at 0 °C (pH = 6.5) and centrifuged at 4 °C at 13,500 rpm for 20 min. Quantities of 10 µL of the supernatant were weighed out to give a reaction mixture with a final volume of 1760 µL, containing 20 µL of orthodianisidine solution (10 mg/mL dissolved in methanol), 30 µL of 0.3% H_2_O_2_ and sodium acetate–acetic acid buffer (pH = 4.5).

Enzyme activity was measured at room temperature for 2 min and absorbance values were recorded every 10 s. To calculate the enzyme activity, the value of the absorbance change during 1 min was multiplied by the dilution of the test sample in the reaction mixture and divided by the molar absorption coefficient of orthodianisidine. The resulting enzyme activity unit U/mL was converted to U/mg using the sample fresh weight.

### 4.8. Proline Level Measurement

The proline content was determined spectrophotometrically. The original method is that of Chinard [79], modified by Ábrahám [80], which shows that at acidic pH, proline forms a red product with ninhydrin. In a first step, 3% sulphosalicylic acid is prepared as follows: 3 g of 5-sulphosalicylic acid is prepared in 100 mL of solution with DV (can be stored freely for weeks). In a second step, acidic ninhydrin is prepared: 0.625 g of ninhydrin, 15 mL of cc. acetic acid, 20 mL of 6 M orthophosphoric acid. After vortexing and heating, it dissolves and the solution can be stored for a few weeks. The method requires 100 mg of plant sample. The plant sample is homogenised with 3% sulphosalicylic acid (5 microl/mg fresh weight), stored frozen, and centrifuged for 5 min at room temperature. The reaction mixture consists of 100 microl of 3% sulphosalicylic acid, 200 microl of cc. acetic acid, 200 microl of acid ninhydrin and 100 microl of sample. The solutions are weighed in test tubes, sealed and kept in a drying oven at 96 °C for 1 h. The reaction is stopped in cold water, then dissolved with 1.5 mL toluene, vortexed for 20 s, and left to stand for 5 min. The absorbance of the supernatant is measured in a glass cuvette at 520 nm. The value obtained is compared with the L-proline standard.

### 4.9. Rhizosphere Measurements

Rhizosphere soil samples were collected from the pots according to Tong et al. [81]. Remaining plant particles were removed with forceps and samples were stored at −20 °C until the analysis. Enzyme activities were measured photometrically at 405 nm, based on the released *p*-nitrophenol. β-glucosidase enzyme activity was calculated from the concentration of *p*-nitrophenol released from *p*-nitrophenyl-β-D-glucoside as a substrate [82]. Alkaline phosphatase activity was measured using the method of Tabatabai and Bremner [83] using *p*-nitrophenyl phosphate for substrate. The substrate used for the measurement of β-glucosaminidase activity was *p*-nitrophenyl-N-acetyl-β -D-glucosaminidase [37].

### 4.10. Statistical Evaulation

The measured data were sorted into a format suitable for statistical evaluation using the Excel data management software package (Office 2021 Professional Plus), and then the IBM SPSS Statistics 29.0.1.0.software package was used for one-way analysis of variance (ANOVA), followed by Duncan test (*p* < 0.05).

## 5. Conclusions

Vegetation is affected by a wide range of stresses individually and in combination with others, which have a major impact on plant vitality. These effects are more pronounced in the urban environment, where air pollution, thermal insulation and higher temperatures, as well as a high proportion of paved surfaces, are all identified as stress factors. Organic biostimulants could decrease the environmental stress effects on horticultural plants. Our aim was to test three biostimulants on *Viburnum opulus* ‘Roseum’ as a popular urban shrub in plant nursery conditions. Our results indicated that the biostimulant Bistep had positive effects on the studied histological and physiological parameters of the test plant; moreover, the measured enzyme activities in the rhizosphere were also higher compared to the other treatments. Biostimulants and plant growth regulators, which are increasingly dominating the sustainable agriculture and horticulture sector, can provide a solution if they are properly selected and applied. These products could help to adapt widely used varieties to stress conditions without compromising their functions and benefits (ornamental value and pollutant sequestration).

## Figures and Tables

**Table 1:** Main characteristics of used biostimulants.

Key Characteristics	Bistep (syn. Ferbanat L)	Kelpak^®^	Yeald Plus
Manufacturer	UAB ALJARA LT-11219 Vilnius, Geniu str. 16-38. Lithuania	KELP PRODUCTS (PTY) LTD 7975 Simon’s Town, Blue Water Close South Africa	De Sangosse Ltd. Hillside Mill Quarry Lane, Swaffham Bulbeck, Cambridge CB5 0LU, UK
Base elements	Microhumates and coupled microelements	Aqueous extracts of Ecklonia maxima	6% nitrogen, 5% zinc, 1% potassium peroxide, 0.03% boron, 0.25% copper, 0.25% iron, 0.25% manganese, 0.001% molybdenum
Effects	Increase soil humus volume, support root development, increase yield and green weight	Support shoot and bud development, support root development	Leaf fertilisation, irrigation, watering
Economic benefit	50% reduction in fertiliser use	It also has a beneficial effect on seed development	Increases root mass by 30–35% in two weeks

## Data Availability

Data is not public due to the measurement is in progress.
